# Aging of mice is associated with p16(Ink4a)- and β-galactosidase-positive macrophage accumulation that can be induced in young mice by senescent cells

**DOI:** 10.18632/aging.100991

**Published:** 2016-07-06

**Authors:** Brandon M. Hall, Vitaly Balan, Anatoli S. Gleiberman, Evguenia Strom, Peter Krasnov, Lauren P. Virtuoso, Elena Rydkina, Slavoljub Vujcic, Karina Balan, Ilya Gitlin, Katerina Leonova, Alexander Polinsky, Olga B. Chernova, Andrei V. Gudkov

**Affiliations:** ^1^ Everon Biosciences, Inc., Buffalo, NY 14203, USA; ^2^ Department of Cell Stress Biology, Roswell Park Cancer Institute, Buffalo, NY, USA

**Keywords:** chronological aging, inflammation, inflammaging, senescence-associated beta-galactosidase, p16INK4a, senescence-associated secretory phenotype, clodronate

## Abstract

Senescent cells (SCs) have been considered a source of age-related chronic sterile systemic inflammation and a target for anti-aging therapies. To understand mechanisms controlling the amount of SCs, we analyzed the phenomenon of rapid clearance of human senescent fibroblasts implanted into SCID mice, which can be overcome when SCs were embedded into alginate beads preventing them from immunocyte attack. To identify putative SC killers, we analyzed the content of cell populations in lavage and capsules formed around the SC-containing beads. One of the major cell types attracted by secretory factors of SCs was a subpopulation of macrophages characterized by *p16(Ink4a)* gene expression and β-galactosidase activity at pH6.0 (β-gal^pH6^), thus resembling SCs. Consistently, *mice with p16(Ink4a) promoter*-driven luciferase, developed bright luminescence of their peritoneal cavity within two weeks following implantation of SCs embedded in alginate beads. p16(Ink4a)/β-gal^pH6^-expressing cells had surface biomarkers of macrophages F4/80 and were sensitive to liposomal clodronate used for the selective killing of cells capable of phagocytosis. At the same time, clodronate failed to kill *bona fide* SCs generated *in vitro* by genotoxic stress. Old mice with elevated proportion of p16(Ink4a)/β-gal^pH6^-positive cells in their tissues demonstrated reduction of both following systemic clodronate treatment, indicating that a significant proportion of cells previously considered to be SCs are actually a subclass of macrophages. These observations point at a significant role of p16(Ink4a)/β-gal^pH6^-positive macrophages in aging, which previously was attributed solely to SCs. They require re-interpretation of the mechanisms underlying rejuvenating effects following eradication of p16(Ink4a)/β-gal^pH6^-positive cells and reconsideration of potential cellular target for anti-aging treatment.

## INTRODUCTION

Understanding the underlying causes of aging in mammals is a prerogative for the rational development of prophylaxis and treatment of this condition and extension of healthy life. Today, multiple theories of aging [1–6] seem to come to an agreement about the pivotal role of sterile chronic systemic inflammation, named “inflammaging” [7,8], as a unifying symptom that develops with age, contributing to development of cancer, metabolic diseases, and other age-related pathologies [9–16]. However, the exact source of inflammaging remains elusive. During the last several years, one specific hypothesis seems to prevail in the field which links aging with the accumulation of SCs. According to this model, *SCs poison tissues with* proinflammatory products of their secretion, a manifestation of a so-called senescence-associated secretory phenotype (SASP) [17–20]. The wide acceptance of the SC hypothesis is based on several studies, all involving genetically modified mice that express specific proteins under control of the *p16(Ink4a)* promoter, believed to be activated in SCs, that enables their selective killing by pharmacological agents [21–23]. Accumulation of p16(Ink4a)-positive cells in tissues of mice occurs with age, and their pharmacological eradication was associated with changes in phenotype consistent with a reduction of biological age and increased longevity in mice genetically prone to accelerated aging [21] or in wild type mice [23], respectively. Eradication of p16(Ink4a)-positive cells was accompanied by the reduction in the proportion of cells within tissues, particularly fat, that express β-gal^pH6^ – one of a few histologically applicable markers of SCs [24]. Thus, accumulation of p16(Ink4a)/β-gal^pH6^-positive cells with age, along with a simultaneous increase of inflammatory factors in tissues was convincingly interpreted as pro-aging activity of SCs.

Cellular senescence can be defined as an epigenetic reprogramming of cells normally capable of proliferation occurring in response to genotoxic (i.e., irradiation, chemotherapeutic drugs, etc.) or oncogenic (activation of dominant oncogenes) stresses [25,26] and characterized by permanent cell cycle arrest, unresolved constitutive DNA damage response and constitutive activation of NF-κB that drives the expression and production of a series of bioactive, largely proinflammatory factors (SASP). Phenomenon of cellular senescence was initially observed and characterized *in vitro* predominantly in human and rodent mesenchymal cells subjected to genotoxic stresses or transduced with oncogenic RAS [27]. The biological sense of senescence has been attributed to cancer prevention by eternal proliferation arrest of cells that could otherwise be dangerous due to their risk of cancer development [28–30]. Numerous attempts to find specific and common biomarkers of senescence resulted in a number of properties, none of which are universal hallmark of SCs. These include already mentioned p16(Ink4a) [31,32], β-gal^pH6^ activity [24,33] and SASP, but also the constitutive presence of signs of DNA damage response, constitutive elevation of p21 and p53, etc. [34–36]. Since the manifestation of many of these traits increases with age, it was reasonably concluded that they are indicative of SC accumulation. However, it remains unclear which particular cells *in vivo* are the carriers of these SC markers.

The SC hypothesis does not provide clear reasons for SC accumulation in old mammals and their absence in young individuals. What is commonly being discussed includes the following scenarios: (i) SC accumulation reflects accumulation of stochastic DNA damage during life; (ii) SC formation is provoked by age-related physiological and metabolic changes leading to the elevation of ROS-mediated genotoxic stress; (iii) SCs result from sporadic and stochastic deregulation of oncogenic pathways in somatic cells with functional p53 and (iv) aging-associated impairment of the immune system function responsible for SC eradication in young organisms [1,5,6,37,38]. However, which of the above assumptions is right, if any, remains to be determined.

In the current study, we address two questions regarding SCs *in vivo*: (i) what is the fate of *in vitro*-generated SCs after their implantation into the body of young mice and (ii) who are the carriers of SC biomarkers *in vivo*? We found that SCs *in vivo* can effectively attract a combination of immunocytes that cause their rapid eradication. A major part of these immunocytes is represented by a subpopulation of macrophages, which display high levels of p16(Ink4a) and β-gal^pH6^ expression, thereby mimicking the most typical properties of SCs. Moreover, a significant part of p16(Ink4a)/β-gal^pH6^-positive cells that accumulated with age in mouse tissues are also represented by macrophages. In light of these observations, re-consideration of the SC hypothesis of aging is discussed.

## RESULTS

### Transplantation model of persistent SCs

**Figure 1:**
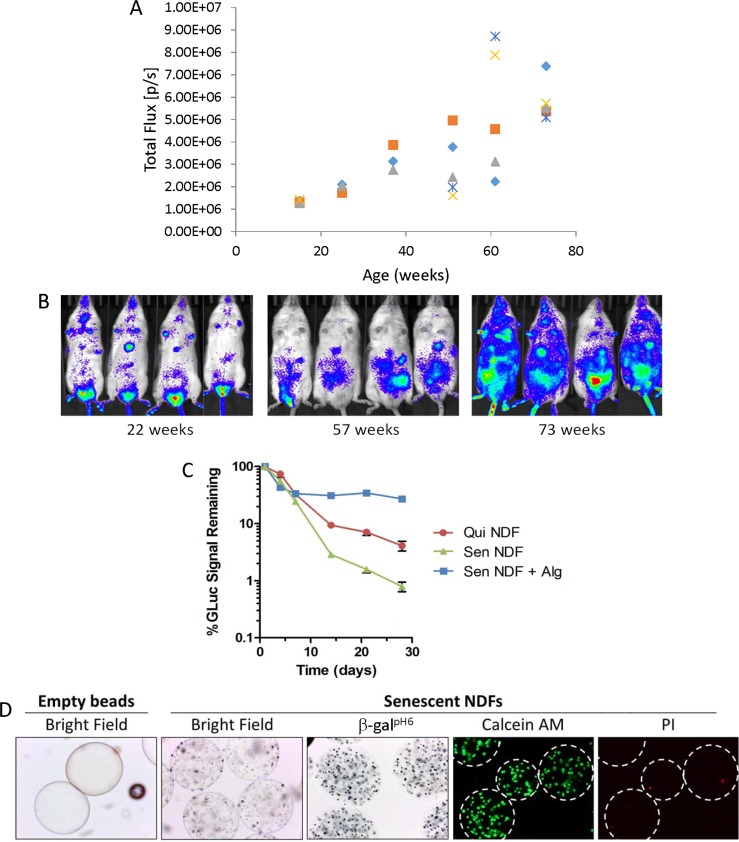
SC implantation *in vivo* (**A-B**) Bioluminescent signal accumulation in a cohort (n=5 mice) of chronologically aged mice harboring a hemizygous p16(Ink4a) knock-in of luciferase (p16^LUC^ mice; p16^Ink4a/Luc^). (**A**) Whole body luminescence (total flux; p/s) for individual mice are depicted. (**B**) Serial bioluminescence imaging of chronologically aged mice. Color scale indicates signal intensity (same thresholds across all time points). (**C-D**) A model of SC implantation into SCID mice. NDF cells harboring a secreted GLuc reporter construct (NDF-GLuc) were implanted intraperitoneally into SCID mice as microcarrier bead cultures that were, prior to injection, cultured in low serum (0.2% FBS) for induction of quiescence (Qui NDF) or irradiated at 20 Gy for induction of senescence (Sen NDF). Alternatively, irradiated NDFs were coated in protective alginate gel (Sen NDF + Alg). Kinetics of NDF-GLuc survival was monitored via measurement of GLuc activity in mouse plasma collected at regular intervals over 28 days. The amount of GLuc activity remaining in the blood over time is expressed as a percentage of activity in plasma 24 hours after cell inoculation. Values depicted are means ± SEM for each group (n = 4-6 mice/group). Differences between all groups are statistically significant after day 7 (p≤0.001). (**D**) Microphotographs of empty alginate beads (no cells) and alginate beads containing embedded irradiation-induced senescent NDFs (bright field images). After embedding senescent NDF cells and before implantation into mice, viability of embedded cells was assessed by labeling live cells with Calcein AM (green) and dead cells with propidium iodide (PI; red), followed by fluorescent microscopy. Senescent NDFs in alginate beads were also assessed for β-gal^pH6^ staining. Representative images are shown (magnification 100x). Successful embedding of cells was indicated by >90% viability (< 10% PI-positive cells).

Senescent cell accumulation with age is thought to be a major source of chronic inflammation underlying age-related diseases [10,20,39]. In fact, the amount of cells expressing SC marker [positive for p16(Ink4a)] gradually increases during mouse life [40] (Fig. 1A,B). However, the reasons why SCs accumulate in tissues with age are not well understood. One of the most obvious explanations is that in young organisms, SCs are cleared more efficiently by the innate immune system [3,41,42]. We sought to investigate the fate and biological effects of human senescent versus non-senescent cells implanted into young severe combined immune deficiency (SCID) mice. Initial development of this model involved generating reporter cells of human neonatal dermal fibroblast (NDF) expressing secreted *Gaussia* luciferase (GLuc), allowing for cell survival to be monitored *in vivo* via measurement of GLuc activity from collected plasma [43,44]. Next, microcarrier bead cultures carrying these GLuc-expressing NDFs (NDF-GLuc) were produced, and cells were made quiescent via serum starvation (0.2% FBS) or senescent via 20 Gy gamma-irradiation. We inoculated 2-3 ×10^6^ cells into the peritoneal cavity of SCID mice via injection of ∼300 μl of packed bead volume, and implanted cell survival via GLuc secretion was monitored from regular blood collections over 28 days (Figure 1C). Mice implanted with quiescent and senescent NDFs exhibited a similar decrease in GLuc signal from plasma up to day 7 post-injection, where 30% of GLuc activity remained compared to the activity observed 24 hours after initial cell implantation. After day 7 however, GLuc activity in mice with SCs decreased at a faster rate than for quiescent cells, suggesting that these SCs are cleared more efficiently. By day 28, less than 1% of the GLuc signal remained from SCs observed on day 1 (a 5.2-fold lower compared to quiescent cells). Faster clearance of SCs than non- SCs suggested either higher fragility of SC cells *in vivo* or the result of activity of a specific mechanism(s) targeting SCs, which presumably involve(s) the innate immune response (since adaptive immunity is compromised in SCID mice).

To discriminate between these possibilities and to identify potential SC killers, we utilized a model where SCs could persist in recipient mice being unreachable for immunocytes. It involves embedding SCs into beads made of alginate, a nanoporous gel that provides a physical barrier from the immune system, while allowing cells inside the beads to exchange nutrients and waste, as well as to release cell secretions, e.g. cytokines [45–47].

Viability of the embedded cells was assessed via Calcein-AM and propidium iodide for the staining of live and dead cells, respectively (Figure 1D). The procedure for generating alginate beads was optimized for >90% SC viability immediately following embedding. In addition, viability was stable during *in vitro* culture prior to inoculation of beads into the peritoneal cavity. Further, the alginate-embedded SCs were further characterized, and determined to be positive for senescence-associated β-galactosidase (β-gal^pH6^) activity (Figure 1D).

Senescent GLuc-expressing NDF were coated in alginate gel beads and injected into SCID mice. GLuc activity was monitored as an indicator of SC viability.

By day 7, a similar loss of GLuc activity was observed compared to unprotected senescent and quiescent NDF-GLuc cells, reflecting a non-selective decrease in transplanted cell numbers likely due to *in vivo* adaptation (Figure 1C). However, unlike the case of bare cells, after day 7 the presence of alginate stabilized GLuc activity observed in plasma, providing a stable signal for up to 3 additional weeks.

The activity of secreted GLuc in mouse plasma indicates that small proteins (GLuc; 25kDa) were reliably released from the alginate beads and systemically detected in peripheral blood. In addition, these initial experiments demonstrated that alginate-coated senescent NDFs survive in the peritoneal cavity for a prolonged time, where they remained functionally active, as inferred by stable GLuc secretion. Protection of SCs from rapid death by embedding in alginate beads strongly argued in favor of the involvement of cellular mechanism in SC eradication *in vivo*. To identify SC killers, we analyzed the content of cells attracted to the peritoneal cavity of mice carrying implanted alginate beads with SCs.

### Nature of cells attracted by SCs into peritoneal cavity

To study immune system components attracted by SC secretions, empty alginate beads or containing 1.5 million senescent NDF were inoculated into mice via intraperitoneal injection and harvested 14 days later, along with the intraperitoneal lavage, for the analysis of cellular content. Since similar results were obtained in SCID, NIH Swiss and C57BL/6 mice, here and below we only show data generated in C57BL/6.

**Figure 2:**
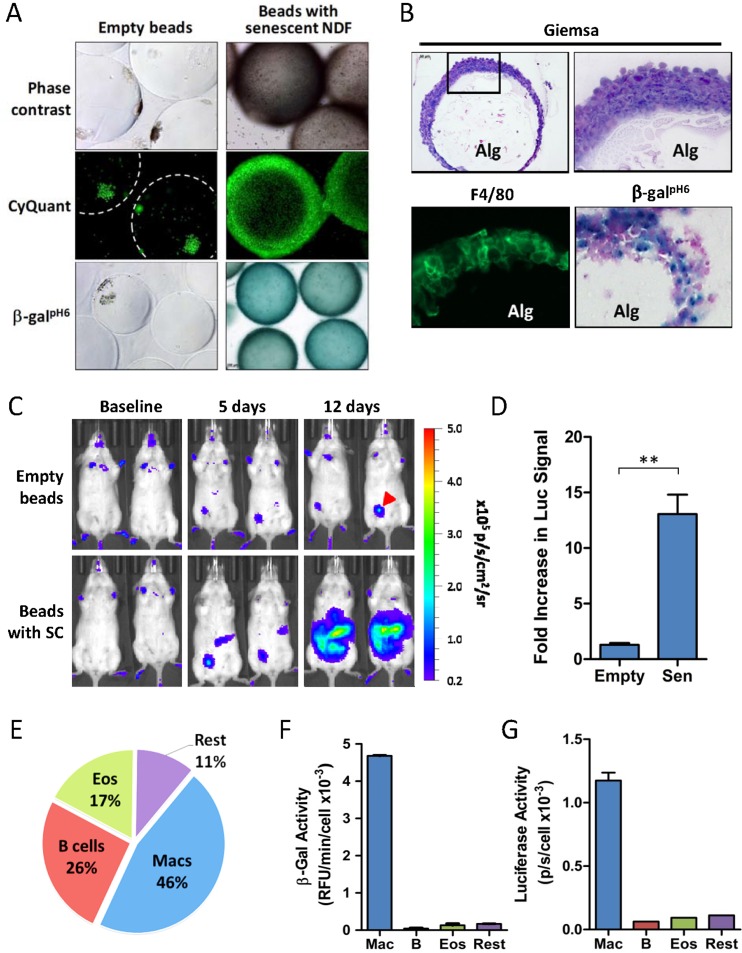
Accumulation of β-gal^pH6^- and p16(Ink4a)-positive immunocytes in response to SC implantation (**A**) Images of whole alginate beads *ex vivo* (either empty or containing SCs) following retrieval from peritoneal cavity of p16^LUC^ mice. The dense encapsulating cell layers surrounding the alginate beads were visualized by phase contrast light microscopy (top panel) or by fluorescent microscopy of samples stained with a DNA dye kit (CyQUANT™ Direct) for visualization of nuclei within live cells (middle panel). β-gal^pH6^ staining reveals activity in cells encapsulating SC-embedded alginate beads (magnification 100x). (**B**) Tissue sections (15-μm) of cryopreserved SC-embedded alginate beads were stained with Geimsa for visualization of histology via light microscopy at 100x and 400x magnification (top left and right panels, respectively), for F4/80 immunofluorescence (green) for visualization of macrophages, showing specific staining of this outer membrane-localized protein (bottom left panel; 400X magnification), and for β-gal^pH6^ activity via X-Gal substrate with nuclear fast red counterstain (400X magnification). Alginate gel containing SCs is indicated (Alg). (**C**) Bioluminescent *in vivo* imaging of p16^LUC^ mice following i.p. inoculation of empty alginate bead (Empty) or alginate-embedded SCs (Sen). Representative serial images acquired two days before bead injection (baseline), and days 5 and 12 after injection, depict increased luminescent signal in mice bearing SCs. The colored scale depicts relative luminescent signal intensity of minimum and maximum thresholds, displayed in terms of radiance. Red arrow indicates injection site wound from alginate bead implantation. (**D**) The amount of bioluminescence on day 12 post-SC injection is expressed as the total flux (p/s) from the abdomen, expressed as the fold increase in signal compared to baseline measurements. (**E**) Analysis of the cell composition of peritoneal lavage from mice bearing SCs collected 2-3 weeks post-inoculation, as analyzed by flow cytometry on live cells immunostained for surface markers. The percent contribution to major cell types is depicted: macrophages (Mac), B lymphocytes (B cells; B), eosinophils (Eos) and remaining cell populations (Rest). This analysis depicts a representative experiment (**E-G**) in which these 4 cell populations were isolated via FACS and assayed for luciferase activity (**F**) and β-gal activity (**G**), normalized to cell number. The gating scheme used for FACS is presented in Supplemental Figure S1. Values depicted are means ± SEM of fold induction for each group (n = 3-6 mice/group).

While empty alginate beads stayed unchanged during 14 days following i.p. injection, SC-containing beads were found to be surrounded by dense capsules formed by multiple layers of cells (Figure 2A and 2B). Enzymatic staining of whole beads using protocol for detection of SCs indicated high β-gal^pH6^ activity in the capsules (Figure 2A). In fact, a large subset of capsule-forming cells stained positive for β-gal^pH6^ (Figure 2B). Further characterization of dissociated capsules showed that they were comprised mostly of macrophages, eosinophils and neutrophils (Supplemental Figure S3B). Immunofluorescent staining of cryosectioned beads revealed that a large portion of cells were F4/80-positive, a widely-accepted marker for mature murine macrophages (Figure 2B). With the large proportion of F4/80- and β-gal^pH6^-positive cells, our data suggests that macrophages are likely to have β-gal^pH6^ activity in the presence of factors released by the beads containing SCs.

The use of p16(Ink4a) reporter mice in several models of senescence and aging demonstrate that the appearance of cells in their tissues positive for β-gal^pH6^ is associated with elevated proportion of p16^Ink4a^-expressing cells. However, the identity of these cells *in vivo* is not well-defined. We sought to test whether β-gal^pH6^-positive immunocytes responding to SCs would be associated with elevated p16^Ink4a^ expression. To do so, alginate-embedded SCs were implanted into young p16^LUC^ reporter mice of the C57BL/6 background (hemizygous knock-in; p16^Ink4a/Luc^), and monitored for p16(Ink4a) induction via increased bioluminescent signal (Figure 2C and 2D). Surprisingly, the presence of SCs induced a 13.1-fold increase in luminescent signal from the abdomen after 12 days *in vivo*. At the same time, no significant increase was observed in mice bearing empty alginate beads, indicating that the response elicited by SCs is not simply due to a foreign body response against alginate beads. Overall, a 10.1-fold increase in luminescence induction was observed between groups bearing empty beads compared to those bearing SCs (p≤0.01). Together, these findings demonstrate that the release of SC secretions from alginate beads strongly induces p16(Ink4a) expression.

In order to identify which cell population(s) contribute to p16(Ink4a) and β-gal^pH6^ signal in response in this model, SC-elicited immunocytes from the peritoneal cavity of p16^LUC^ mice were collected 2 weeks after SC inoculation. FACS analysis of live-stained peritoneal infiltrates revealed cells to be of hematopoietic origin (≥90% CD45^+^) and comprised of three main cell types (constituting ∼75% of lavage). These cells were sorted into four populations for subsequent determination of luciferase and β-gal^pH6^ activity: 1) B lymphocytes (CD19^+^; ∼26%), 2) eosinophils (CD19^−^ CD11b^+^ CD170^+^; ∼17%), 3) macrophages (CD19^−^ CD11b^+^ F4/80^+^; ∼46%), and 4) a pool of remaining cell populations (11%), including neutrophils (CD11b^+^ Ly-6G^+^), CD11b^−^ cells (e.g., T lymphocytes) and CD11b^+^ F4/80^−^ cells (e.g. monocytes, NK cells) (Figure 2E; Supplemental Figure S1). Analysis of cell lysates from these sorted populations revealed a substantial enrichment of both markers in macrophages but not in other cell types (Figure 2F and 2G). In fact, macrophages were the only cell population to provide a detectable luciferase activity, which was more than 6-fold higher per cell than other populations (Figure 2F). Similarly, macrophages were more 10.5-fold enriched for β-gal^pH6^ activity per cell (Figure 2G). Given their abundance, these data suggest that macrophages almost exclusively contribute to the luminescent signal and β-gal^pH6^ activity from SC-elicited infiltrates found within the peritoneal lavage.

### Elevated p16 and β-gal^pH6^ expression in SC-elicited macrophages

**Figure 3:**
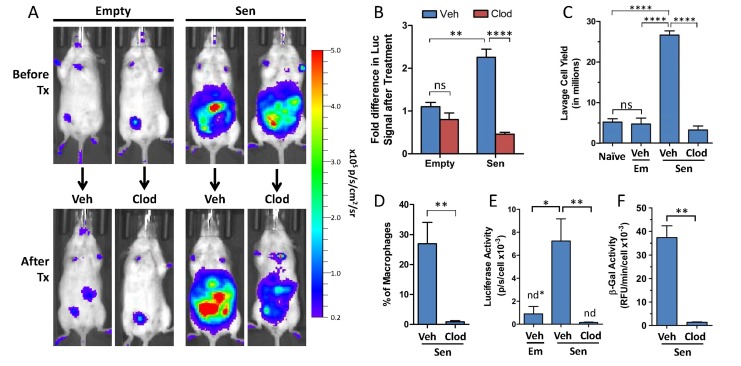
Pharmacological clearance of macrophages *in vivo* depletes luciferase and β-gal^pH6^ activity from p16^LUC^ mice bearing SCs (**A**) Representative serial images depicting *in vivo* bioluminescence from p16^LUC^ mice acquired 12 days after inoculation of empty beads (Empty) or alginate-embedded SCs (before treatment) and one week later (after treatment; 18-20 days post-inoculation) after two i.p. administrations of liposomes containing PBS control (Veh) or clodronate (Clod). Colored scale depicts relative luminescent signal intensity of minimum and maximum thresholds, displayed in terms of radiance. (**B**) The amount of luminescence (total flux; p/s) from the abdomen after treatment is expressed as the fold difference compared to the signal measured before treatment for each group. (**C**) Total yield of cells recovered from peritoneal lavage from naïve mice, of liposomal vehicle-treated mice bearing empty beads (Em/Veh), or of liposomal vehicle- or clodronate-treated mice bearing SCs (Sen/Veh and Sen/Clod, respectively). (**D**) The amount of macrophages present in peritoneal lavage of treated mice bearing SCs is expressed as the percentage of F4/80-positive cells present within the population of live CD45-positive cells, as assessed via flow cytometry on immunostained cells. Cell lysates of whole lavage after treatment were assayed for luciferase activity (**E**) and β-gal^pH6^ activity (**F**), normalized to cell number. Values depicted are means +/− SEM (n = 3-7 mice/group). ns = not statistically significant, p>0.05; n.d. = not detectable, values depicted indicate detection limit (defined as 2-fold above background reading) per cell number analyzed.

We next sought to determine the contribution of p16(Ink4a)/β-gal^pH6^-positive macrophages to the increased bioluminescence from p16^LUC^ mice bearing alginate beads containing SCs. To do so, mice were treated intraperitoneally with a liposomal formulation of clodronate, a reagent commonly used for selective depletion of phagocytes (e.g. macrophages) [48,49]. For mice bearing alginate-embedded SCs treated with vehicle control (empty liposomes in PBS), signal continued to increase 2.3-fold compared to the pre-treatment measurements (Figure 3A and 3B). In contrast, clodronate treatment resulted in a 2.2-fold decrease in bioluminescence. Thus, the combined effect of clodronate treatment was a 5.1-fold decrease in bioluminescent signal from p16^LUC^ mice compared to the vehicle-treated group (p≤0.0001). In mice bearing empty beads, which do not induce p16(Ink4a) (Figure 2C), a non-significant decrease was observed upon clodronate treatment. These data suggest that cells with phagocytic activity, i.e. macrophages, are responsible for the majority of p16(Ink4a) induction in response to senescent NDFs. This finding is consistent with our previous observation that macrophages from lavage are the single contributing source of luciferase activity.

To confirm that the loss of *in vivo* bioluminescence in mice upon treatment with clodronate is a result of macrophage clearance, peritoneal lavage from mice bearing alginate-embedded SCs was collected for analysis following administration of vehicle or clodronate liposomes. First, the effects of clodronate treatment on cell yields from peritoneal lavage were determined. While no difference in lavage cell yields was observed between naïve mice and vehicle-treated mice bearing empty alginate beads (∼5×10^6^ cells), for vehicle-treated mice inoculated with alginate-embedded SCs, a large number of cells infiltrated into the peritoneal cavity (2.6×10^7^ cells; a >5-fold increase in cell yield over the group with empty beads; p≤0.0001) (Figure 3C). Clodronate treatment of mice bearing alginate-embedded SCs resulted in decreased yields from peritoneal lavage, with cell counts similar to control groups (3.2×10^6^ cells).

Next, the clearance of macrophages from clodronate-treated mice was confirmed in mice bearing alginate-embedded SCs, where flow cytometric analysis showed that the proportion of F4/80^+^ cells in lavage was reduced to less than 1% following clodronate treatment (Figure 3D). This is consistent with our observation that clodronate treatment effectively depleted F4/80-positive cells from the spleens of these mice (Supplemental Figure S2A and S2B). To assess the impact of clodronate treatment on the presence of luciferase and β-gal^pH6^ activity in mice bearing SCs, cell lysates from peritoneal lavage infiltrates were assayed. Luciferase activity was found to be undetectable from the lavage of all mice that received clodronate, a >47-fold reduction between vehicle- and clodronate-treated groups (p≤0.01) (Figure 3E). Loss of luciferase activity from the lavage of clodronate-treated mice was accompanied by a concomitant decrease in β-gal^pH6^ activity, a 37-fold reduction compared to vehicle-treated mice (p≤0.01) (Figure 3F). Thus, consistent with analysis from sorted cell populations from lavage, *in vivo* clodronate treatment of mice bearing alginate-embedded SCs resulted in the depletion of p16(Ink4a)/β-gal^pH6^ -positive macrophages from the peritoneal cavity.

Clodronate treatment efficiently depleted a major portion of luminescent signal measured *in vivo* (∼80% reduction compared to vehicle-treated). However, residual luminescent signal remained, approximately 6-fold over initial baseline measurements (i.e. prior to SC injection). Thus, we sought to identify additional sources of luciferase signal *in vivo*. Histological analysis of alginate beads from vehicle-and clodronate-treated mice bearing SCs revealed that clodronate was inefficient at eliminating F4/80-positive cells from the cells encapsulating the alginate beads, suggesting that liposomes may be incapable of accessing inner cell layers of the capsule. (Supplemental Figure S3A). Cells dissociated from alginate beads were analyzed on flow cytometry to determine cell composition, confirming that clodronate was unable to exert a significant effect on the composition of encapsulating cells (Supplemental Figure S3B). Consistent with our previous observations that macrophages were the single greatest contributor to luciferase and β-gal^pH6^ activity from peritoneal lavage, the inability of clodronate to successfully deplete macrophages from alginate beads was associated with unaltered levels of luciferase and β-gal^pH6^ activity measured from lysates (Supplemental Figure 3D and 3E). Taken together, this data strongly suggests that the bioluminescent signal remaining *in vivo* following clodronate treatment is due to the continued presence of macrophages surrounding alginate beads.

### Clodronate liposomes depletes p16(Ink4a)- and β-gal^pH6^-positive cells in chronologically aged mice

**Figure 4:**
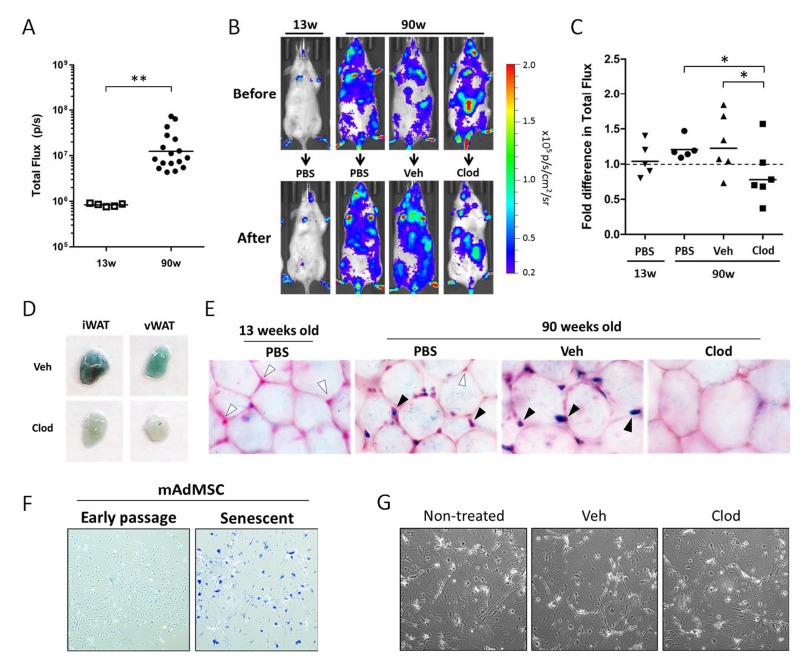
Clodronate treatment depletes p16(Ink4a)-positive and β-gal^pH6^-positive cells from chronologically aged p16^LUC^ mice (**A**) Bioluminescent baseline readings from the abdomen of young (13 weeks) versus old (90 weeks) p16^LUC^ mice (n=5 and 17 mice/group, respectively). Geometric mean is depicted on graph. (**B-C)** Old mice were randomized among 3 groups based on bioluminescence from the abdomen (n=5-6 per group): treatment with PBS, vehicle liposomes in PBS (Veh), or liposomal clodronate (Clod). Bioluminescence of the abdomens was measured after two clodronate treatments (i.p., three days prior and i.v., one days prior to luminescent measurement). (**B**) Representative serial images of p16^LUC^ mice depicting luminescence (in radiance) before and after treatment regimen. Colored scale depicts relative luminescent signal intensity of minimum and maximum thresholds, displayed in terms of radiance. (**C**) The amount of luminescent signal (total flux; p/s) from the abdomen of treated p16^LUC^ mice is expressed as the fold difference compared to measurement before treatment. Geometric mean is depicted on graph. (**D**) Inguinal and visceral (perigonadal) depots of white adipose tissue (iWAT and vWAT, respectively) were collected from vehicle and clodronate liposome treated 90-week old p16^LUC^ mice and stained for β-gal^pH6^ activity. Representative photographic images are presented. (**E**) Representative light microscopy images (magnification, 200x) of β-gal^pH6^-stained visceral adipose tissue counterstained with nuclear fast red. Cells residing between adipocytes (indicated by the presence of nuclear stain) are β-gal^pH6^-negative (white arrow) or -positive (black arrow). These cells are altogether absent from large regions in clodronate-treated mice (as depicted). (**F**) Representative images of β-gal^pH6^-stained cultures of mouse adipose-derived mesenchymal stromal cells (mAdMSC) from p16^LUC^ mice at early passage (p1 cultures) or 10 days after 20Gy gamma-irradiation (Senescent). SCs stain positive for β-gal^pH6^ and are enlarged and morphologically distinct from early passage. (**G**) Phase contrast light microscopy images of senescent mAdMSCs following overnight (20 hr) with 50 μg/mL clodronate liposomes (Clod), or similar dilution (1:100) of vehicle liposomes (Veh) or PBS (non-treated), indicating no observable cell death or effects on these cells.

Our data demonstrate that macrophages, through increased number and/or activation state, are capable of generating a bioluminescent signal easily detectable via bioluminescent imaging. We next evaluated whether macrophages contribute to the increased luminescent signal in chronologically aged p16^LUC^ mice *in vivo*. Bioluminescent signal from of 90-week old p16^LUC^ mice was 9.0- fold higher than 13-week old mice (Figure 4A). To determine if macrophages contribute to this increased signal, bioluminescence was measured before and after phagocyte depletion via clodronate liposomes (Figure 4B and 4C). Mice were randomized across treatment groups based on baseline measurements of signal intensity. Since it is not known how ingestion of vehicle liposomes may affect bioluminescence in phagocytes in old p16^LUC^ mice, both PBS alone and vehicle liposomes (in PBS) were administered as controls. On average, clodronate-treatment resulted in a 1.5-fold difference in p16(Ink4a) expression between both control groups of old mice (p≤0.05). The percent of old mice with >20% decrease in luciferase activity following treatment was 0% and 17% for PBS and vehicle liposome groups, respectively, compared to 67% of mice in the clodronate-treated group. These findings demonstrate that phagocytes contribute to p16(Ink4a) promoter-driven reporter signal in chronologically aged mice.

Adipose tissue is thought to be a major source of SCs and chronic inflammation in chronologically aged mice [15,23]. Old mice possess depots of white adipose tissue that stain positive for β-gal^pH6^ activity compared to young mice [23]. To determine whether macrophages contribute to the pool of β-gal^pH6^-positive cells in adipose tissue of chronologically aged mice, 90-week old p16^LUC^ mice were treated with liposomes containing PBS (vehicle) or clodronate, and inguinal and visceral (perigonadal) depots of white adipose tissues were collected for analysis. Whole pieces of fat stained for β-gal^pH6^ activity revealed that that clearance of phagocytes resulted in a strong reduction in staining in clodronate-treated mice compared to vehicle-treated (Figure 4D). Similar results were obtained after treatment of aged wild type mice of the same background and opposite gender (male C57BL/6; 64 weeks old).

Clodronate liposomes have been shown to deplete macrophages from various depots of white adipose tissues in mice [48,49]. To confirm that *in vivo* clodronate treatment was successful in clearing β-gal^pH6^-positive cells, β-gal^pH6^-stained fat was analyzed via light microscopy following nuclear counterstain (Figure 4E). In 90-week old mice treated with PBS as a control, we observed the presence of β-gal^pH6^-positive cells *in situ*, interspersed between mature adipocytes in visceral adipose tissue. In contrast, in adipose tissue of young mice (13 weeks old) treated with PBS, cells were present in the same location within the adipose tissue (assessed via nuclear stain), but these cells were negative for β-gal^pH6^ staining. These results confirmed that β-gal^pH6^-positive cells in adipose tissue accumulated with age. The treatment of old mice with vehicle liposomes had little effect on the presence of β-gal^pH6^-positive cells interspersed between adipocytes; however, these cells were stained more intensely compared to PBS-treated aged mice. Unlike the other three groups, treatment with clodronate resulted in the absence of both β-gal^pH6^-positive cells and nuclear staining between mature adipocytes, indicating that these cells were effectively cleared by clodronate. Further, cells expressing elevated levels of β-galactosidase (assessed immunofluorescent staining by anti-GLB1 antibodies) were found to be F4/80-positive (Supplemental Figure 4). The presence of both markers was also reduced following clodronate treatment. Together, our data indicate that macrophages residing in visceral adipose tissue of old mice are predominantly β-galactosidase-positive.

Recent literature attributes the age-dependent increase in β-gal^pH6^-positive cells in adipose tissue to the appearance of senescent pre-adipocytes [23]. However, we demonstrate that phagocytic cells contribute to the vast majority of β-gal^pH6^ staining in chronologically aged mice. To assess whether senescent adipose-derived mesenchymal stromal cells possess sufficient phagocytic activity to be cleared by clodronate, we first established primary cultures from the adipose-derived stromal vascular fraction of p16^LUC^ mice. After the first passage of these cultures, minimal macrophage contamination was observed (<5%). Senescence induction of primary cells *in vitro* (20 Gy gamma-irradiation followed by 10 days in culture) gave rise to common features of SCs, including loss of proliferation, enlarged morphology, and positive β-gal^pH6^ staining (Figure 4F). In addition these cultures displayed a 9.7-fold increase (p<0.0001) in p16(Ink4a) promoter-driven luciferase activity from early passage cultures (352 ± 17.1 p/s/cell x10^3^) to senescent cultures (3,404 ± 89.8 p/s/cell x10^3^). However, *in vitro* treatment of SC cultures with liposomal clodronate had no effect on the appearance or viability of these SC cultures, nor an effect on luciferase activity (Figure 4G), as compared to non-treated and vehicle liposome-treated SC cultures. As expected, this same dose of clodronate efficiently cleared adherence-selected peritoneal macrophages from naïve mice (data not shown). The mechanism of clodronate-dependent clearance of β-gal^pH6^-positive cells requires a sufficient level of phagocytic activity, a phenotype not previously associated with senescent stroma. These data strongly suggest that the β-gal^pH6^-positive cells observed in adipose tissue in situ are macrophages and/or other professional phagocytes.

## DISCUSSION

Pharmacological elimination of p16(Ink4a)/β-gal^pH6^-positive cells in mice (both genetically predisposed to accelerated aging [21] and wild type [23]), resulted in improved physiological performance consistent with rejuvenation (reduction of biological age). Since both represent well-known traits of SCs, these observations were convincingly interpreted as arguments in favor of the SC hypothesis [39,50,51] and greatly stimulated studies aimed at generation and use of senolytic compounds as potential anti-aging drugs [52–56]. Here we demonstrate that the same combination of traits can be acquired by macrophages *in vivo* following stimulation with unidentified factors produced by SCs embedded in alginate beads. Furthermore, a significant proportion of p16(Ink4a)/β-gal^pH6^-positive cells accumulated in mouse tissues *in vivo* during chronological aging also have properties of macrophages since they can be selectively killed by clodronate liposomes [49,57,58], indicative of their phagocytic capability that is not a characteristic of SCs.

Although these data do not contradict the claimed role of SCs in aging, they call for a significant modification of this hypothesis. They point at a new type of cells, p16(Ink4a)/β-gal^pH6^-positive macrophages, which also accumulate with age and were likely pharmacologically eradicated from the organisms of old mice in the experiments conducted by Baker et al. [21,23] and Demaria et al. [22]. Hence, this subpopulation of macrophages has the same right as SCs to be considered a possible contributor to aging. Obviously, the relative impact of either of these cell types to age-related inflammation remains to be determined.

**Figure 5:**
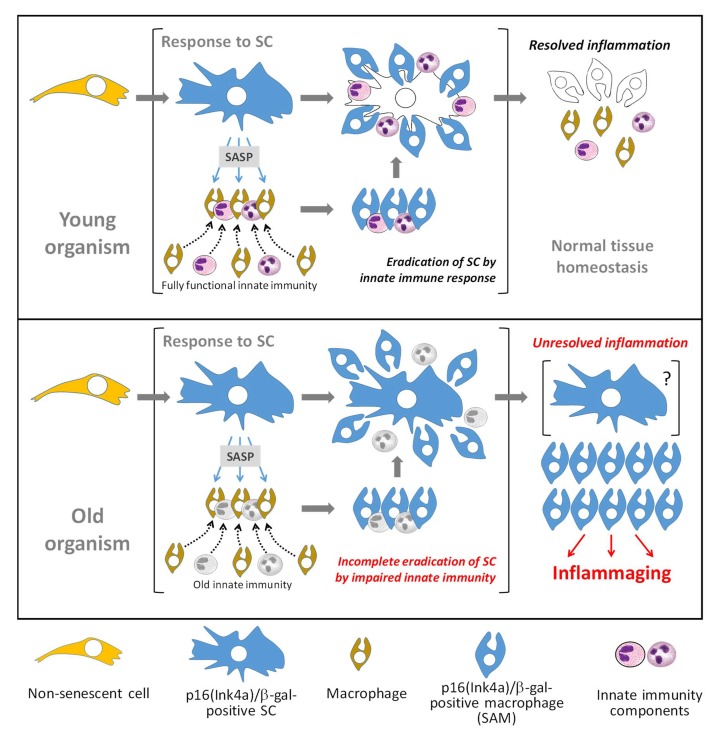
Schematic of hypothetical model of *in vivo* accumulation of p16(Ink4a)/β-gal^pH6^-positive cells in naturally aged organisms In young mammals (top panel), the secretion of SASP by p16(Ink4a)/β-gal^pH6^-positive SCs facilitates the attraction of innate immune components necessary for efficient targeting and destruction of SCs. SC secretions activate recruited macrophages, inducing a p16(Ink4a)/β-gal^pH6^-positive phenotype in them. After the successful eradication of SCs, inflammatory factors subside and tissue homeostasis resumes. This resolution results in the loss of p16(Ink4a)/β-gal^pH6^-positive cells from the tissue, as macrophages with this phenotype are cleared or discharge their activated state. However, in old animals (bottom panel), impairments in innate immunity result in the inability to efficiently recognize or destroy SCs. This results in establishment of chronic, inflammation induced by products of secretion of SCs and SC-associated macrophages (SAM). Accumulation of SAMs can be a manifestation of unresolved innate immune response leading to chronic sterile systemic inflammation typical for aged organisms.

The main findings of the current study are incorporated into a hypothetical model presented in Figure 5. Rapid elimination of *in vitro*-generated SCs implanted i.p. into mice was interpreted as the activity of a specific immunological mechanism of recognition and elimination of such cells. Consistently, multiple cellular components of innate immunity, involving macro-phages, eosinophils, and lymphocytes, concentrate within the peritoneal cavity of mice implanted with SC-containing alginate beads, presumably representing the SC “killers” attracted to their “victims” through products of SC secretion. This suggests that actively secreted (SASP) and other factors released from the SC-containing alginate beads are part of “find-me” and “eat-me” signals that attracts immunocytes to SCs, similar to the clearance of dying cells [3,59–62].

Earlier reports that described the role of neutrophils and NK cells in SC elimination [41,42,63,64] seemingly contradict our observations on the contents of immuno-cytes attracted by SCs. This apparent controversy can be explained by differences in the experimental conditions: the above-mentioned reports were predominantly focused on early stages of SC recognition and killing, while we characterized the content of cells attracted by SC secretion at much later times – more than 10 days following SC implantation. Our experimental setup enabled prolonged release of bioactive molecules from immunoisolated SCs. This uniquely allowed us to observe p16(Ink4a)/β-gal^pH6^-expressing macrophage accumulation in the peritoneal cavity through their constant attraction and/or phenotypic conversion.

Remarkably, elevated activity of β-galactosidase was earlier described as a sign of murine peritoneal macrophage maturation *in vivo* and *in vitro* [65,66].

Macrophages were shown to upregulate β-galactosidase activity in the presence of apoptotic cells, where the intensity of endogenous β-galactosidase activity, even when assayed at pH 7.3, was found to be a confounding source of signal in an *in vivo* model of p53-responsive bacterial LacZ reporter following irradiation [67]. Unlike senescence, switch of macrophages to β-galactosidase-expressing phenotype is independent of p53, as macrophages in p53^−/−^ mice exhibit β-galactosidase activity in regions of apoptosis [68]. However, β-galactosidase activity is not a marker of any type of macrophage activation since thiolglycollate-elicited or bacteria-activated macrophages show a decrease in β-gal expression [65].

p16(Ink4a) induction in macrophages is also not uniquely attributed to exposure to SCs. p16(Ink4a) protein has been implicated in macrophage polarization and activation of proinflammatory signaling: *in vitro* non-senescent IFN-γ polarized M1 macrophages expressed higher levels of p16(Ink4a) than IL-4-polarized human M2 macrophages. During *in vitro* differentiation of bone marrow-derived macrophages, p16(Ink4a) protein expression increased in parallel with induction of F4/80 marker. However, this increase did not influence the cell cycle distribution of differentiating cells. Similar polarizing properties were obtained with primary macrophages upon ectopic p16(Ink4a) expression [69,70]. Murakami et al. [71] recently showed that p16(Ink4a) regulates degradation of IRAK1 and consequent secretion of IL-6 in mouse and human macrophages but not fibroblasts. In addition, another senescence-associated tumor suppressor p19ARF has been reported as a new p53-independent regulator of inflammatory response in macrophages modulating TLRs signaling [72,73]. Hence, the induction of the senescence-associated macrophage (SAM) phenotype may not specifically require SCs, but rather represent a specific type of macrophage activation or differentiation in response to certain natural physiological stimuli. It remains unclear whether accumulation of p16(Ink4a)/γ-gal^pH6^-positive macrophages in tissues of old mice depends on SCs. Regardless of the nature of p16(Ink4a)/γ-gal^pH6^-positive macrophages, their presence in much higher numbers in tissues of old mice compared to young, presumably reflecting either constitutive presence of activating factors in old animals or age-associated defects in their “discharging” (returning to prior phenotype) or elimination. The latter possibility is shown in Figure 5. In either case, accumulation of SAMs can be a manifestation of unresolved innate immune response leading to chronic sterile systemic inflammation typical for aged organisms.

Positive physiological consequences of pharmacological eradication of p16(Ink4a)/γ-gal^pH6^-positive cells in mice [21,23] convincingly define such cells as a likely source of inflammaging and a target for treatment and prophylaxis of aging. Our findings do not shake this exciting conclusion but rather suggest reconsidering the nature of such cells and, therefore, modifying the focus of anti-aging drug discovery: from SC-targeting senolytic compounds to agents capable of targeting other p16(Ink4a)/γ-gal^pH6^-positive cells, such as the subpopulation of macrophages, SAMs, described in our work. In fact, the indications of anti-aging activity recently reported for senolytic compounds [52,55,56,74] were different and less pronounced than those described by Baker et al. in mice following eradication of p16(Ink4a)-positive cells [21,23] and may reflect an exaggerated view on SCs as the sole source of inflammaging.

## MATERIALS AND METHODS

### Cell culture

Primary human neonatal dermal fibroblasts (NDFs; AllCells, LLC) were pooled equally from three separate donors. NDFs were maintained in Dulbecco's modified Eagle Medium (DMEM) with phenol red supplemented with 10% (v/v) FBS (Gibco; Grand Island, NY), 100 units/mL of penicillin, 100 μg/mL of streptomycin and 2 mM L-glutamine, and 1X MEM non-essential amino acids. Cells were cultured in a tissue culture incubator at 37°C and 5% CO_2_. NDFs were maintained at <80% confluency by serial passage after enzymatic dissociation via TrypLE (Thermo Fisher Scientific). GLuc-expressing NDF cells (NDF-GLuc) were puromycin-selected following lentiviral transduction of a PGK1 promoter-driven reporter construct that was synthesized and cloned into pRSIT vector by Genscript (Piscataway, NY). This construct constitutively expresses secreted *Gaussia princeps* luciferase (“8990” mutant [75]), v5-tagged Histone H3, and puromycin from the same transcript, separated by T2A sequences. NDFs were routinely tested for mycoplasma, and confirmed to be negative using the MycoAlert mycoplasma detection kit (Lonza).

Primary cultures of adipose-derived mesenchymal stromal cells (mAdMSC) were established from the stromal vascular fraction of inguinal adipose tissues of young p16^LUC^ mice, as described [76]. Isolated adherent cultures were maintained in DMEM/F12 medium supplemented with 15% FBS and 1X anti-biotic/anti-mycotic solution (Thermo Fisher Scientific) in a tri-gas tissue culture incubator at 37°C, 5% CO_2_ and 3% O_2_. Medium was change twice per week, and cell passaging performed when cells reached ∼80-90% confluency. For induction of senescence, passage 1 cells were irradiated in suspension at 20 Gy, and replated cultures were maintained at 21% O_2_ and 5% CO_2_.

### Animals

Male and female C57BL/6J mice with hemizygous p16(Ink4a) knock-in of firefly luciferase (p16^Ink4a/Luc^) were obtained from our breeding colony, originally obtained from Dr. Normal E. Sharpless [40]. Male C57BL/6J wild type mice were obtained from Jackson Laboratories (Bar Harbor, ME), female NIH Swiss mice [Cr:NIH(S)] were obtained from Charles River (Wilmington, MA), and male C.B-Igh-1blcrTac-Prkdcscid/Ros mice (SCID) were obtained from the animal facility at the Roswell Park Cancer Institute (Buffalo, NY).

Animals were provided a commercial rodent diet (5% 7012 Teklad LM-485 Mouse/Rat Sterilized Diet, Harlan) and sterile drinking water ad libitum. All the animals were confined to a limited access facility with environmentally-controlled housing conditions through-out the entire study period and maintained at 18-26°C, 30-70 % air humidity, 12-h light/dark cycle. The animals were housed in micro-isolation cages under pathogen-free conditions, and if necessary, acclimatized in the housing conditions for at least 5 days prior to the start of the experiment. Animal usage in this experiment was approved under Institutional Animal Care and Use Committee (IACUC) at the Roswell Park Cancer Institute.

### Microcarrier bead culture

Dry CytoPore™ 2 beads (GE Healthcare; Marlborough, MA ) were hydrated and sterilized in 70% ethanol for 15 minutes, washed in PBS, and incubated with complete medium containing 10% FBS overnight at 4°C. Cells were attached to these modified cellulose-based macroporous microcarrier beads by incubating with freshly lifted NDF cell suspensions at a ratio of 5 million cells to 1 mL of packed, hydrated bead volume. Microcarrier bead culture was performed using complete DMEM medium in 250-mL tissue culture-grade vented cap Erlenmeyer flasks at 20-40 mL per 1 mL of hydrated packed bead volume, placed on a rotatory shaker within a tissue culture incubator. Three days after inoculation of CytoPore™ 2 beads, cells were switched into complete medium containing 0.2% FBS (quiescent condition), or exposed to 20 Gy gamma-irradiation via radioactive Cs source (Shepherd MK I-68 gamma-irradiator) (senescent condition), and maintained in culture for an addition 3-4 days, up to 2 weeks with media changed every 3-4 days.

For embedding of irradiated NDF microcarrier cultures into alginate beads, 400 μl of cytopore beads were mixed with 600 μl of 3% alginate solution. After thorough mixing, the suspension was loaded into a 1-mL syringe and mounted to an infusion pump (5-mm). The alginate encapsulation procedure was based on a gas-driven mono-jet device positioned 24-cm above the 100 mM SrCl_2_ gelling solution, which was continuously stirred via magnet stir bar (700 rpm). The suspension NDF microcarrier cultures in alginate was sprayed into the gelling solution at an infusion rate of 0.8 mL/min and air flow rate of between 6-9 L/min. Alginate-coated beads were incubated in the gelling solution for 5 minutes, followed by thorough washing in PBS and transfer to warm complete medium. Beads were incubated on shaker in tissue culture incubator for at least 24 hours prior to injection.

### Encapsulation of SCs in alginate beads

Ultra-pure low-viscosity (20-200 mPas) sodium alginate (PRONOVA UP LVG) powder was purchased from NovaMatrix (Sandvika, Norway). Alginate powder was dissolved in a 1% (w/v) mannitol solution to make a 3% (w/v) solution of sodium alginate. The final solution was filter-sterilized (0.2 μm) and stored at 4°C. A 100 mM solution of strontium chloride (SrCl_2_) (Sigma) dissolved in sterile water was used for alginate gelation. NDF cells were synchronized (switch to 0.2% FBS at high confluency overnight) prior to irradiating cells in suspension at 20Gy. Cells were then re-plated at subconfluent densities in complete medium (10% FBS) for at least 3 days. Irradiated NDF cells were lifted, washed in PBS, and re-suspended in 100 μl of saline. This cell suspension was then mixed with the 3% alginate solution (0.9mL), and after thorough mixing, immediately loaded into a 1-mL syringe for extrusion. The alginate encapsulation procedure was based on a gas-driven mono-jet device positioned 14-cm above the 100 mM SrCl_2_ gelling solution, which was continuously stirred via magnet stir bar (125 rpm). The suspension of NDF cells in alginate was sprayed into the gelling solution at an infusion rate of 0.6 mL/min and air flow rate of 7 L/min. Alginate-coated beads were incubated in the gelling solution for 5 minutes, followed by thorough washing in PBS and transfer to warm complete medium. Beads were incubated on shaker in tissue culture incubator for at least 24 hours prior to injection. Viability of senescent NDF cells embedded in alginate beads was verified by Calcein AM (Thermo Fisher Scientific) / propidium iodide (Sigma) staining of live/dead cells.

### Implantation of senescent NDFs

Microcarrier bead cultures of NDF cells (with or without alginate coating) were washed three times in neat RPMI medium (Gibco; Grand Island, NY) and approximately 300 μl of packed bead volume (2 to 3 million cells) were injected intraperitoneally into isoflurane-anesthetized mice via a 16-gauge needle. Since SCs can be directly embedded into alginate, there was no need to pre-establish cells on microcarrier beads prior to alginate encapsulation. Further, direct embedding generated more uniform-sized beads for studying effects of immune system response. SC-embedded alginate beads (cells suspended in alginate) were injected similar to microcarrier beads.

### Clodronate treatment

Liposomal clodronate (Clodrosome®; 5 mg/mL) and liposomal vehicle in PBS (Encapsome®) were obtained from Encapsula NanoSciences (Brentwood, TN). These liposomal formulations or PBS control (as indicated) were administered to mice as 200 μl injections per mouse (20-40 mg/kg), either intraperitoneally or intravenously. Injections were administered twice per mouse, 2-4 days apart. For experiments utilizing alginate beads, mice were administered two intraperitoneal injections, with collection of lavage and alginate beads 1-2 days after the last injection. For treatment of old p16^LUC^ mice, mice received the first injection intraperitoneally, and the second injection intravenously via tail vein was administered 2 days later. Adipose tissues (inguinal or perigonadal visceral fat) were collected from mice 1-2 days after the last treatment.

For *in vitro* treatment of clodronate and related controls, cell cultures were washed once with medium, then incubated with medium supplemented with a 1:100 dilution of liposomal clodronate suspension (50 μg/mL clodronate), liposomal PBS control or D-PBS control (non-treated). Cells were maintained for 20 hours at standard conditions during incubation with treatments. Plates were then washed twice with D-PBS and photographed. Cells were then lifted for determination of luciferase activity.

### Bioluminescent imaging

Mice were injected intraperitoneally with a 200 μl solution of 15 mg/mL D-luciferin potassium salt (Syd Labs; Boston, MA) in D-PBS without calcium and magnesium. At 10 minutes post-injection, isoflurane-anesthetized mice were placed into the IVIS Spectrum *in vivo* bioluminescent imaging system (PerkinElmer; Waltham, MA) for detection of luciferase activity (60-second exposure). Bioluminescence in p16^LUC^ mice was quantified as total flux (p/s) of luminescent signal from the abdomen using via Living Image® software.

### Collection of peritoneal lavage and alginate beads

Two- to three-weeks post-implantation of alginate beads into the peritoneal cavity, peritoneal lavage and free alginate beads were collected from CO_2_-asphyxiated mice. The skin covering the abdomen was dissected away, revealing the intact peritoneal cavity. Mice were then injected i.p. with 7 mL of 2% heat-inactivated FBS in saline using a 27-gauge needle. After gently massaging the abdomen, a 25-gauge needle was used to collect the peritoneal lavage. Lavages were then stored on ice prior to preparation of cell lysates. The cell density of collected peritoneal lavage was measured using Via1-Cassette™, where live and dead counts of nucleated cells from lavage were quantitated on a NucleoCounter® NC-200 (ChemoMetec; Allerod, Denmark) via acridine orange and DAPI staining. The peritoneal lavage was then pelleted (400 x g for 5 minutes at 4°C), re-suspended in BD Pharm Lyse lysing buffer (diluted to 1X in sterile, double-distilled water) purchased from BD Biosciences (San Jose, CA), and incubated in the dark at room temperature for 7 minutes. Three volumes of complete medium were added before pelleting cells and re-suspending in PBS.

For collection of alginate beads, the wall of the abdomen was opened. Beads were then flushed from the peritoneal cavity with saline containing 2% heat-inactivated FBS. Beads were then washed several times in PBS prior to analysis. Mouse immunocytes encapsulating the alginate beads were stained with CyQuant® Direct Cell Proliferation Assay (Thermo Fisher Scientific), and the stained nuclei from viable immunocytes were imaged via fluorescent microscopy.

### Preparation of cellular lysates

Cell suspensions of lavage were recounted after RBC lysis, and equal numbers of cells were transferred to Eppendorf tubes for centrifugation (400xg for 5 minutes at 4°C). Cell lysates of peritoneal lavage were obtained by re-suspending equal numbers of lavage cells or *ex vivo* alginate beads into 1X Reporter Lysis Buffer (Promega) supplemented with 0.5% Triton X-100 (Sigma) and 1:100 Dilution of protease inhibitor cocktail (Sigma). Lysis was performed at room temperature for 5 minutes with agitation, and the samples were then returned to ice. Using gel loading pipette tips, the cell extract was removed from alginate beads and placed into a clean Eppendorf tube. Lysates were clarified via centrifugation (16,000 x g for 10 minutes at 4°C) and stored on ice until use in enzymatic assays. Protein concentration of cell lysates was measured using the Pierce BCA Protein Assay Kit (Thermo Fisher Scientific; Waltham, MA) per the manufacturer's instructions.

### Flow cytometry

Before staining, mouse peritoneal lavage cells were treated with BD Pharm Lyse lysing buffer (diluted to 1X in sterile, double-distilled water) for red blood cell lysis, then washed and resuspended in flow cytometry staining buffer (eBioscience; San Diego, CA). Cells were released from alginate bead capsules using enzymatic dissociation reagent, TrypLE. After blocking with anti–CD16/CD32 antibodies (clone 93, eBioscience) for ten minutes the cells were stained with the following fluorochrome-conjugated antibodies to surface receptors in an 8-color staining combination: FITC-labeled anti-Ly-6G (1A8, Miltenyi Biotec); V500-labeled CD11b (M1/70, BD Horizon), and antibodies from eBioscience: PE-labeled anti-CD335 (29A1.4); PE/Cy5.5-labeled anti-CD19 (eBio1D3); PerCp-eFluor710-labeled anti-CD170 (1RNM44N); APC-labeled anti-CD45.2 (104); APC-eFuor780 labeled F4/80 (BMB); eFluor450-labeled anti-Ly-6C (HK1.4). After 30 minutes incubation on ice in the dark, cells were washed with flow cytometry staining buffer and resuspended in the same buffer. To distinguish dead cells, impermeable DNA stain Bobo3 (Molecular Probes, Eugene, Oregon) was added to the cell suspension (20 nM final concentration) three minutes before acquisition. All sorting and analysis experiments were performed on Roswell Park Cancer Institute FACS facility custom instruments from BD Immunocytometry systems (FACSAria I or LSRII, respectively) using BD FACS Diva Software (BD Biosciences). Data were collected for 0.2 to 1 ×10^6^ cells and analyzed with FCS Express 4 (De Novo Software; Glendale, CA). To distinguish autofluorescent cells from cells expressing low levels of individual surface markers (in case of CD11b, Ly-6G, F4/80 and CD335 markers), we established upper thresholds for autofluorescence by staining samples with fluorescence-minus-one control stain sets in which a reagent for a channel of interest is omitted. Compensation was performed using single-color controls prepared with OneComp beads (eBioscience), or single-stained cell suspensions and calculated either with FACS Diva Software (in case of sorting) or with FCS Express (for composition analysis).

### Immunofluorescence

Tissue samples were placed into plastic molds filled with NEG-50 frozen section medium (Fisher) and snap-frozen in a slurry of 2-methylbutane and dry ice. Fresh-frozen sections 12-μm thick were cut on a CM1900 cryotome (Leica), placed on Histobond slides (StatsLab), dried for 15 min and kept at −20°C until staining. Before staining, slides were warmed up to room temperature, fixed 5 min with 4% formaldehyde in PBS, and washed 3 times with PBS. Sections were incubated with block solution (5% normal donkey serum, 0.25% triton x-100, PBS) and incubated with rat monoclonal anti-F4/80 antibody conjugated with AlexaFluor488 (BioLegend, 1:50 dilution); rat monoclonal anti-B220 antibody conjugated with Cy5 (eBioscience, 1:50 dilution); and mouse monoclonal anti-smooth muscle actin (SMA) antibody conjugated with Cy3 (Sigma, 1:1000 dilution). All antibodies were diluted in block solution. Stained sections were mounted with ProLong Diamond anti-fade reagent with DAPI (Invitrogen). For staining of whole fat, tissue was first fixed for 5 hours in 4% paraformaldehyde at 4°C. The adipose tissue was then washed in PBS, incubated for 1 hour in blocking solution, and stained for 4 hours with rabbit polyclonal anti-GLB1 (Abcam, 1:200 dilution) followed by detection via donkey anti-rabbit IgG secondary antibody conjugated with AlexaFluor488 (Jackson ImmunoResearch, 2 ug/ml for 2 hours), and rat monoclonal anti-F4/80 antibody conjugated with AlexaFluor488 (BioLegend, 1:50 dilution). Immunostained tissue was incubated in 80% fructose overnight following nuclear counterstain with Hoechst 33342 (0.5μg/ml) for 15 min. Stained sections and whole tissues were analyzed under AxioImager Z1 microscope equipped with epi-fluorescence and AxioCam MRm digital camera (Carl Zeiss Inc.). Images were captured and processed with AxioVision software (Carl Zeiss Inc., release 4.5.3).

### Firefly luciferase assay

Luciferase activity of cell lysates was accessed using Bright-Glo™ Luciferase Assay System (Promega; Madison, WI) according to manufacturer's instructions with minor modifications. Briefly, cell lysates (as described previously) or cell suspensions in D-PBS were added to a 96-well white plate (OptiPlate-96, Perkin Elmer), followed by the addition of an equal volume of 2x reconstituted Bright-Glo™ Assay Reagent. Acquisition of the luminescent signal was performed on the Infinite® M1000 PRO microplate reader (Tecan; Männedorf, Switzerland). The mean maximum signal from technical replicates was used to estimate the luciferase activity for each sample. Background readings were measured after incubation of equal volumes of 2x luciferase reagent with either D-PBS or lysis buffer (as appropriate). Background signal was subtracted from sample signal, and normalized either by cell number or protein content per reaction. The ratio of background signal to sample signal (signal-to-noise ratio) was calculated for each sample. Sample signals less than 2-fold above background were considered to not be reliably detectable. To estimate detection threshold for a given sample, the background signal was normalized to cell number or protein amount in the reaction.

### *Gaussia* luciferase assay

Blood was collected from the saphenous vein (50 μL) into heparinized collection vials (Sarstedt; Nümbrecht, Germany) at regular intervals (twice per week, up to 28 days) from SCID mice bearing NDF cells transduced with a GLuc reporter construct. Plasma was obtained following centrifugation at 10,000 x g for 7 minutes at 4°C, and stored at −80°C with no substantial loss of signal. For measurement of GLuc activity, plasma was diluted 1:10 in PBS containing 0.1% Triton X-100 in a solid white 96-well microplate. Next, two volumes of a 100 μM solution of coelenterazine in 1X PBS supplemented with 0.3M ascorbic acid and 0.1% Triton X100, pH 7.5, was added to the diluted plasma sample and luminescent signal was measured immediately on a microplate lumino-meter (Tecan) using a 500 ms integration time. Coelenterazine was obtained as a dry powder from NanoLight Technologies (Pinetop, AZ); stock solutions at 5 mg/ml in acidified ethanol were stored at −80°C.

### Determination of β-gal^pH6^ activity

β-gal^pH6^ activity of cryosectioned fresh frozen tissue or whole fat tissue was assayed as previously described [77]. Briefly, cryosections were fixed for 5 minutes with 2% (v/v) para-formaldehyde and 0.2% (v/v) glutaraldehyde, washed in PBS, and incubated with 0.1% (v/v) X-Gal in 100 mM citric acid/sodium phosphate buffer (pH 6.0), containing 2 mM MgCl_2_, 150 mM NaCl, 5 mM K_3_Fe(CN)_6_, 5 mM K_4_Fe(CN)_6_, and 0.02% (v/v) NP-40 at 37°C, and monitored for development of X-Gal product for up to 18 hours. The reaction was stopped by washing slides in PBS, followed by counterstaining with nuclear fast red (Ricca Chemical Company; Arlington, TX). Visceral and inguinal fat was processed similarly.

A fluorogenic substrate for β-galactosidase, 4-MUG (4-methylumbelliferyl β-D-galactopyranoside; Sigma-Aldrich), was used for the quantitation of enzymatic β-gal activity from cell lysates, assayed under β-galpH6 conditions (i.e. pH 6.0). In a 96-well white microplate, 5 μl of diluted cell lysate was combined with 45 μl of fluorogenic β-galpH6 reaction buffer: 1 mM 4-MUG in 100 mM citric acid/sodium phosphate buffer, pH 6.0, containing 2 mM MgCl2, 150 mM NaCl and 0.02% NP-40. Kinetics of product formation at room temperature was determined by measuring fluorescence (Ex/Em 360nm/440nm) of the reaction mixture at regular intervals over a 2-hour time period. Reaction endpoints were determined by addition of 150 μl of 700 mM sodium carbonate. The rate of reaction (RFU/min) for each sample was determined, and normalized per μg of protein.

### Statistical analysis

All statistical analyses were performed using GraphPad Prism version 5.00 (GraphPad Software, San Diego, CA). Statistical comparison of two groups was performed using an unpaired students' two-tailed t-test. For statistical comparisons involving more than two groups, a one-way analysis of variance (ANOVA) with Bonferroni post-hoc test or a Kruskal-Wallis one-way ANOVA with Dunn's post-hoc test (for non-parametric data) was performed to determine differences between groups. Differences were considered statistically significant at p-values less than 0.05: not significant (ns), p > 0.05; *, p≤ 0.05; **, p≤0.01; ***, p≤0.001; ****, p≤0.0001. All data presented as mean ± standard error.

## SUPPLEMENTAL DATA FIGURES



## Abbreviations

SC: senescent cell
SASP: senescence-associated secretory phenotype
β-gal^pH6^: β-galactosidase activity detectable at pH6.0, a marker of SCs
FACS: fluorescence-activated cell sorting
GLuc: Gaussia princeps luciferase
i.p.: intraperitoneal
mAdMSC: mouse adipose-derived mesenchymal stromal cells
NDF: neonatal dermal fibroblasts
NK: natural killer cells
PGK: phosphoglycerate kinase
SCID: severe combined immune deficiency
