# Supplementary material for: Aging of mice is associated with p16(Ink4a)- and β-galactosidase-positive macrophage accumulation that can be induced in young mice by senescent cells
Source: Aging (Albany NY). 2016 Jul 6;8(7):1294–311. doi: 10.18632/aging.100991 (PMC4993332; doi:10.18632/aging.100991)
Supplement: Supplementary file 1 [file aging-08-1294-s001.pdf]

SUPPLEMENTAL DATA

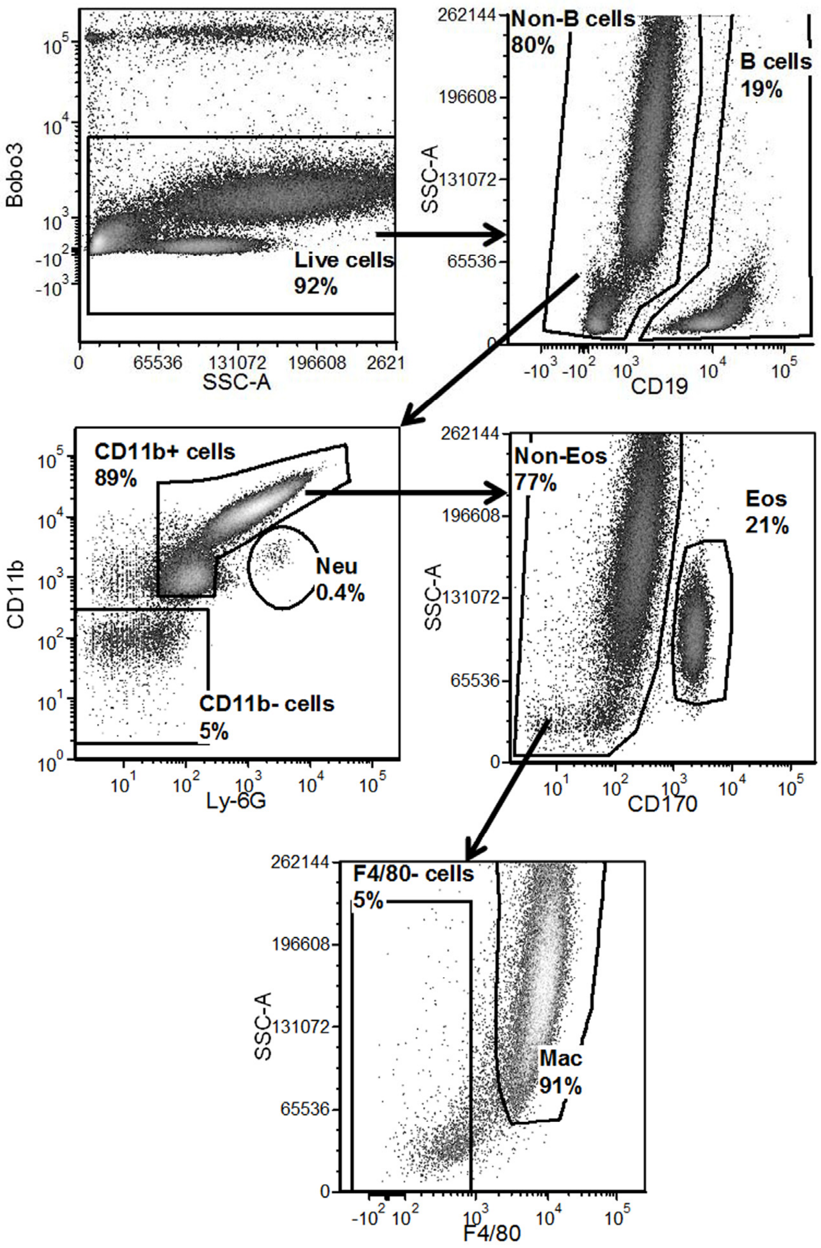

Figure S1. Flow cytometry gating scheme for analysis and isolation of immunocyte populations via FACS. Gating scheme used to identify and sort populations of immune cells from peritoneal lavage and from cells detached from alginate beads containing senescent cells (SCs) (see materials and methods). Representative analysis of peritoneal lavage from mice responding to alginate embedded SCs used for FACS sorting of B lymphocytes (B cells), eosinophils (Eos), macrophages (Mac), and remaining cell populations (Neu, CD11b-negative cells, F4/80-negative cells).

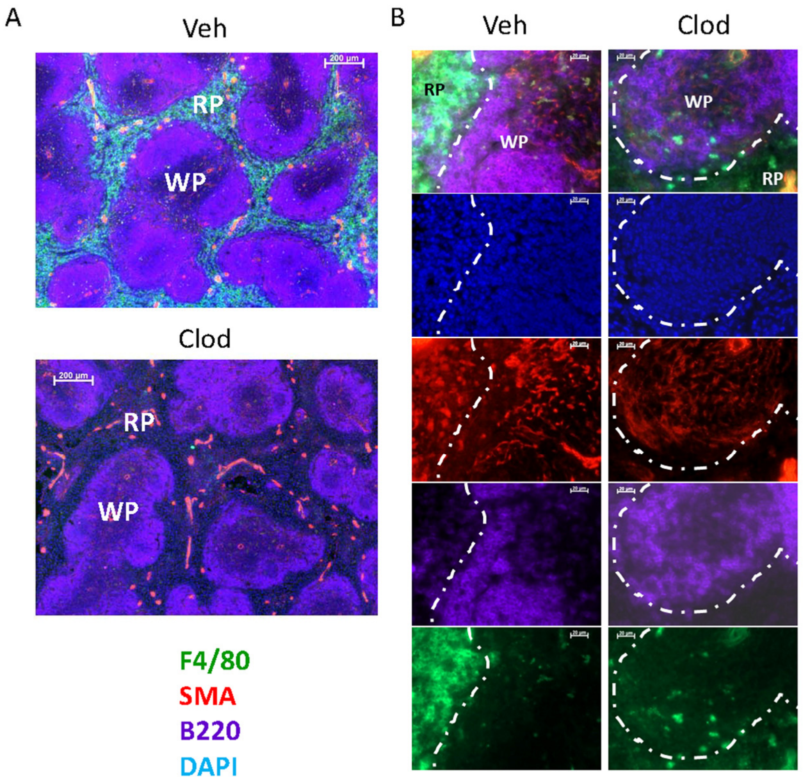

Figure S2. Liposomal clodronate depletes F4/80-positive cells from mouse spleen. Fluorescence microscopy images of immunofluorescent staining of spleens collected from mice after treatment with liposomal formulations of vehicle or clodronate at (**A**) 100X or (**B**) 400X magnification: macrophage marker (anti-F4/80, green; highlighting areas of 'red pulp'), stromal/fibroblast marker (anti-SMA), B lymphocyte marker (anti-B220, purple; highlighting areas of 'white pulp') and counterstained for nuclei with DAPI (blue).

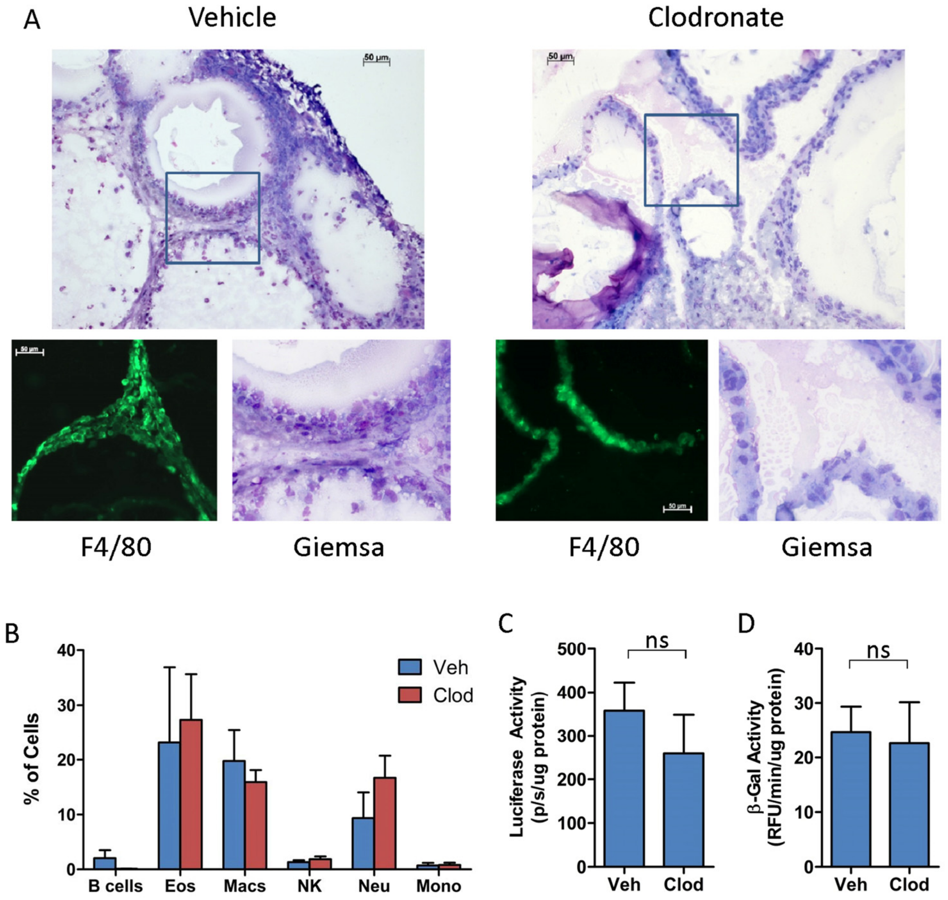

Figure S3. Clodronate treatment inefficiently clears F4/80-positive cells encapsulating alginate-embedded SCs. (**A**) Microscopy images of *ex vivo* alginate-embedded SCs from $p16^{LUC}$ mice treated twice i.p. (3 days apart) with liposomal formulations of vehicle PBS (left panel) and clodronate (right panel), and collected on day 19 (one day after the last treatment). Cryosectioned alginate beads were stained for Geimsa and visualized via light microscopy at 100X magnification (top panels) and 400X magnification (bottom right panels). Images of anti-F4/80 immunostaining are also presented (bottom left panels; green). (**B**) Encapsulated beads from mice with or without clodronate administration were treated with enzymatic reagents for release of attached cells, followed by immunostaining for surface markers on live cells for quantitation of the following cell types via flow cytometry (see Supplemental Figure S1 for gating scheme): B lymphocytes, B cells; eosinophils, Eos; macrophages, Macs; natural killer cells, NK; neutrophils, Neu; and monocytes, Mono. Cells lysates obtained directly from alginate beads *ex vivo* were assayed for luciferase activity (**C**) and $\beta\text{-gal}^{pH6}$ activity (**D**), normalized per µg of protein. Values depicted are means ± SEM (n=3-6 mice/group).

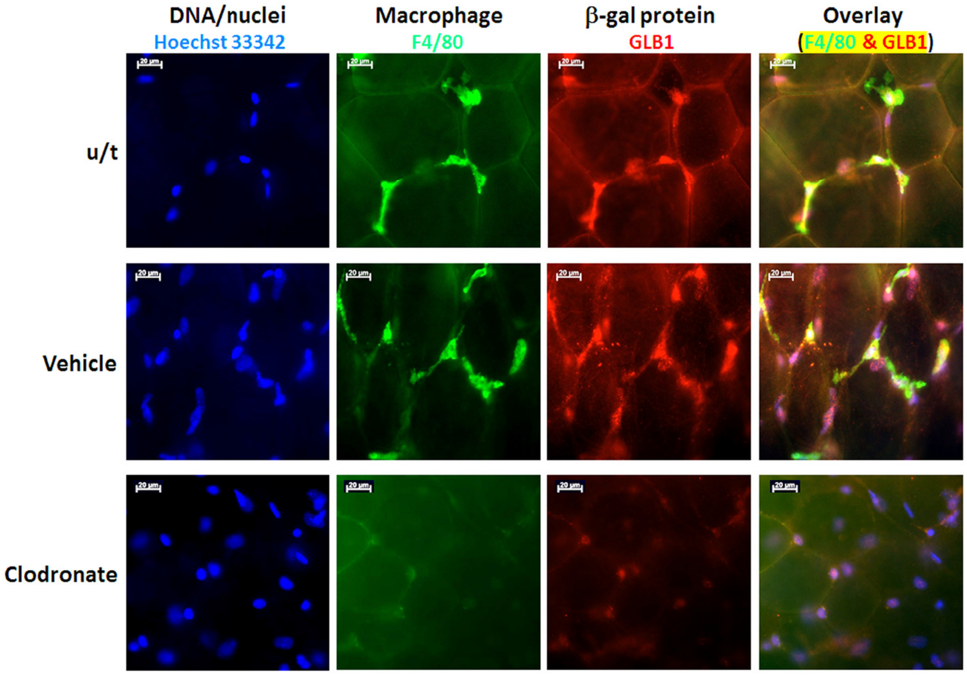

Figure S4. Liposomal clodronate depletes GLB1- and F4/80-positive cells from visceral adipose tissue of aged mice. Visceral adipose tissue from aged C57Bl/6 mice (53-weeks old) treated with PBS, vehicle liposomes (Veh), or liposomal clodronate (Clod) were stained for macrophage marker (anti-F4/80; green), beta-galactosidase (anti-GLB1; red) and nuclear counterstain (Hoechst 33342; blue). Fluorescence microscopy images are presented (magnification, 400x).
